# Can artificial intelligence improve the diagnosis and prognosis of disorders of consciousness? A scoping review

**DOI:** 10.3389/frai.2025.1608778

**Published:** 2025-05-30

**Authors:** Mirjam Bonanno, Davide Cardile, Piergiuseppe Liuzzi, Antonio Celesti, Giuseppe Micali, Francesco Corallo, Angelo Quartarone, Francesco Tomaiuolo, Rocco Salvatore Calabrò

**Affiliations:** ^1^IRCCS Centro Neurolesi “Bonino-Pulejo”, Messina, Italy; ^2^Department of Mathematics and Computer Sciences, Physical Sciences and Earth Sciences, University of Messina, Messina, Italy; ^3^Department of Health Sciences, 'Magna Graecia' University of Catanzaro, Catanzaro, Italy; ^4^IRCCS Fondazione Don Carlo Gnocchi ONLUS, Firenze, Italy; ^5^Scuola Superiore Sant'Anna, Istituto di BioRobotica, Pontedera, Italy; ^6^Department of Biomedical, Dental Sciences and Morphological and Functional Images, University of Messina, Messina, Italy; ^7^Department of Clinical and Experimental Medicine, University of Messina, Messina, Italy

**Keywords:** artificial intelligence, machine learning, deep learning, interpretability, diagnosis, prognosis, neurorehabilitation, disorder of consciousness

## Abstract

**Background:**

Artificial intelligence (AI), in the form of machine learning (ML) or deep learning (DL) models, can aid clinicians in the diagnostic process and/or in the prognosis of critically medical conditions, as for patients with a disorder of consciousness (DoC), in which both aspects are particularly challenging. DoC is a category of neurological impairments that are mainly caused by severe acquired brain injury, like ischemic or hemorrhagic strokes or traumatic injuries. The aim of this scoping review is to map the literature on the role of ML and DL in the field of diagnosis and prognosis of DoCs.

**Materials and methods:**

A scoping search, started from 3rd October 2024, was conducted for all peer-reviewed articles published from 2000 to 2024, using the following databases: PubMed, Embase, Scopus and Cochrane Library.

**Results:**

We found a total of 49,417 articles. After duplicate removal and title/abstract screening, 613 articles met the inclusion criteria, but 592 articles were excluded after full-text review. Therefore, only 21 studies involving DoC subjects were included in the review synthesis.

**Conclusion:**

Advancing AI in the field of DoC requires standardized data protocols and consideration of demographic variations. AI could enhance diagnosis, prognosis, and differentiation between states like unresponsive wakefulness syndrome (UWS) and minimally conscious state (MCS). Additionally, AI-based applications personalize rehabilitation by identifying key recovery factors, optimizing patient outcomes.

## 1 Introduction

Disorder of consciousness (DoC) is a category of neurological impairments that are mainly caused by severe acquired brain injury, like ischemic or hemorrhagic stroke or traumatic injury (Molteni et al., 2023). From a behavioral perspective, DoCs are divided into two main clinical states: the vegetative state that is recently named unresponsive wakefulness syndrome—UWS, in which the patient regains a sleep–wake cycle although he or she is not aware of him/herself or of the surrounding environment; and the minimally conscious state (MCS) in which patients partially reacquire a certain degree of awareness. MCS patients can further classified into two categories called MCS plus (+) and MCS minus (−). These two categories are distinct from each other for their clinical characteristics: MCS+ tends to manifest specific pivotal behaviors, such as consistent and reproducible movement to command, while MCS− tends to manifest automatic motor responses (Bodart et al., 2013). One of the challenges in diagnosing and classifying DoCs is related to determine the presence or absence of a residual state of consciousness following severe acquired brain injury. This state, referred to as “covert consciousness, “is observed in DoC bedridden patients who are unable to exhibit overt signs of residual consciousness (Yang et al., 2024). Since this takes the form of a brain activation in the absence of a behavioral response to simple commands, according to Edlow et al. (2021), covert consciousness can be also defined as cognitive-motor dissociation, and it can be detected by specific neuroimaging and neurophysiological techniques (Edlow et al., 2021). Among these techniques, resting-state functional magnetic resonance imaging (rs-fMRI) is notable for being more accessible and objective than task-based fMRI, as it does not depend on auditory or visual stimuli, removing the need for active patient participation (Mwansisya et al., 2017), which is not always possible in this patient population. An increasing number of studies using rs-fMRI have employed traditional machine learning (ML) methods to explore neuroimaging biomarkers for differentiating patients with varying levels of consciousness (Campbell et al., 2020). Although these findings represent promising strides in elucidating differences in brain activity between DoC patients and controls, the precise differentiation of DoC subgroups, such as UWS and MCS, and between MCS+ or MCS− remains a challenge. A second critical aspect of DoC management is the lack of therapeutically opportunities to integrate rehabilitation management. In this sense, understanding the key prognostic factors that influence patients’ recovery can provide valuable insights into determining the most effective and personalized treatment for this patient population. To this end, the use of artificial intelligence (AI) and related technologies (e.g., machine learning and deep learning) are increasingly becoming popular. For example, the application of AI within the diagnostic process supporting clinicians could be of great value for the healthcare context and the patients’ overall wellbeing (Poalelungi et al., 2023). The use of AI in the rehabilitation field could be useful to monitor patients’ progress over time and to adapt the treatment according to the specific patients’ needs. Other authors have already investigated the role of AI in the context of dementia (Andargoli et al., 2024). These authors suggested that AI can support clinical decision, providing an opportunity for a better and earlier diagnosis, an improved diagnostic accuracy, and management of people living with dementia. Similarly, Abdelrahim et al. (2025), investigated the role of AI applied to non-invasive neuroimaging techniques for the early diagnosis of patients with autism spectrum disorder. The authors stated that AI was highly beneficial in classifying patients according to the different diagnosis and to distinguish the different severity levels. In the field of DoC, the integration of AI in clinical management could be useful to understand the biomarkers associated with recovery and potential treatments that lead to better outcomes. By applying various algorithms and techniques, machine learning (ML) processes, analyzes, and improves data to enhance the accuracy of its predictions (Sarker, 2021). In the clinical context, supervised and unsupervised ML algorithms are more commonly used. However, they may present various limitations, such as potentially lower accuracy in highly complex, multivariate problems, but they can be applied to smaller sample sizes. This aspect is fundamental since DoCs are neurological conditions with different etiologies, making them extremely heterogeneous. On the other hand, Deep learning (DL), a subset of ML, offers significantly higher accuracy due to the greater number of hyperparameters (Pugliese et al., 2021; Kufel et al., 2023; Zhao et al., 2023). Clinically, DL applications were mostly used to process neuroimaging, predicting neurological disorders through magnetic resonance imaging (MRI) scans (Miotto et al., 2017). In this way, these technologies could aid clinicians in the diagnostic process and in the rehabilitation pathway, especially in the field of DoC, in which this aspect is particularly challenging.

The aim of this scoping review is to map the literature on the role of AI, with regards to ML and DL, in the field of diagnosis and prognosis of DoCs, highlighting the current strengths and gaps, suggesting future perspectives. In particular, we tried to answer to the following questions:

1. What is the role of AI in the diagnosis and prognosis of DoCs?
2. What are the most used AI technologies in the field of DoCs?
3. How can AI improve patients’ care?

## 2 Materials and methods

To address the research questions, a comprehensive search was conducted across key research databases, including PuMed, Scopus, Embase and Cochrane library, to identify relevant studies. Using predefined inclusion and exclusion criteria, multiple reviewers independently screened the studies at each stage of the review process. Any disagreements were resolved through consensus, after which the relevant data were extracted for analysis to help answer the research question. Following the formulation of the research questions, the review process involved several key steps: identifying relevant studies, selecting studies, extracting and organizing data, and summarizing and reporting the findings.

This scoping review followed the Preferred Reporting Items for Scoping Reviews-Systematic Reviews and Meta-Analyses (SR-PRISMA) guidelines (Page et al., 2021) to enhance the transparency, completeness, reliability, and validity of the reported information (SR-PRISMA checklist is available in the Supplementary Material). The protocol was registered in Open Science Framework (OSF): https://doi.org/10.17605/OSF.IO/6D89Z.

### 2.1 PICO model

We defined our combination of search terms using a PICO (Eriksen and Frandsen, 2018) (population, intervention, comparison, outcome) model. The population was limited to patients with DoC including patients with coma, vegetative state, minimally conscious state and post-traumatic confusional state. The intervention included all studies who explored, described or applied AI to diagnosis, prognosis and clinical management of these disorders. The comparison was related to the differences between AI models, ML and DL algorithms. The results included any contribution to the diagnosis, prognosis and clinical management of DoC or AI potential impact on clinical decision.

### 2.2 Eligibility criteria, search strategy and research databases

A scoping search, started from 3trd October 2024, was conducted for all peer-reviewed articles published from 2000 to 2024, using the following databases: PubMed, Embase, Scopus and Cochrane Library, which are the most used databases in the context of medicine and bioengineering field. We used a common search query for each consulted database: (((((((artificial intelligence [Title/Abstract]) OR (deep learning [Title/Abstract])) OR (machine learning [Title/Abstract])) AND (disorder of consciousness [Title/Abstract])) OR (coma [Title/Abstract])) OR (vegetative state [Title/Abstract])) OR (minimally conscious state [Title/Abstract])) OR (post-traumatic confusional state [Title/Abstract]).

We included all studies on the adult population (>18 years) affected by DoCs, including UWS and MCS, from any etiology (e.g., cerebro-vascular impairments and/or traumatic brain injury—TBI). Specifically, the inclusion criteria were: (i) patients with DoCs; (ii) Use of AI and related technologies for the diagnostic and prognostic assessment of patients with DoC; (iii) written in English language; and (iv) published in a peer-reviewed journal.

Articles describing theoretical models, methodological approaches, algorithms, and basic technical descriptions were excluded. We excluded also: (i) animal studies; (ii) conference proceedings and review; (iii) studies involving children; (iv) case reports. The list of articles was refined for relevance, revised, and summarized, with key topics identified based on the inclusion and exclusion criteria. Given the limited literature available, various study designs were included in the qualitative synthesis: (i) Randomized Controlled Trials (RCTs); (ii) Observational studies; (iii) Cross-sectional studies; (iv) Case–control studies; and (v) Cohort studies.

### 2.3 Selection process

To reduce the risk of bias, the review process was conducted under strict blinding conditions. Two independent reviewers (MB and DC) were blinded to the identities and affiliations of the authors during both the screening and data extraction stages. This blinding aimed to minimize potential biases, such as publication or language bias. All search results were imported into an online database (RYYAN) (Ouzzani et al., 2016), where the reviewers independently evaluated each study’s relevance. After an initial screening based on titles and abstracts, the blind was lifted, and any disagreements on article inclusion or exclusion were resolved through discussion between the two reviewers.

### 2.4 Data extraction and data items

Following full-text selection, data extraction from the included studies was recorded in a data sheet. The extracted information included: assigned ID number, study title, year of publication or presentation, first author, study aim, sample size, baseline characteristics, type of intervention/evaluation and control, intervention setting, type of neurophysiological and clinical predictors included, type of ML/DL/AI algorithm used, results and performance metrics (e.g., accuracy, sensitivity, specificity, area under the curve—AuC), and presence of interpretability techniques.

## 3 Results

The initial electronic data search yielded a total of 49,417 potentially relevant articles on PubMed, Embase, Web of Science and Cochrane Library. Of these, 17,184 were duplicated and 77 were non-English articles. A total of 31,543 studies were excluded due to title or abstract. Of the resulting 613 articles, 21 fully met the inclusion criteria and were therefore included in this review. The 21 included studies involved a total of 14,683 patients and 180 healthy controls (reported in only 9 studies). Specifically, 6 studies included 11,256 patients with DoC without specifying the type of consciousness clinical state; 9 studies reported on 367 patients with UWS and 329 with MCS; and 6 studies included 2.731 patients in a comatose state.

See Supplementary Table 1 for a complete summary of the studies included.

**Figure 1:**
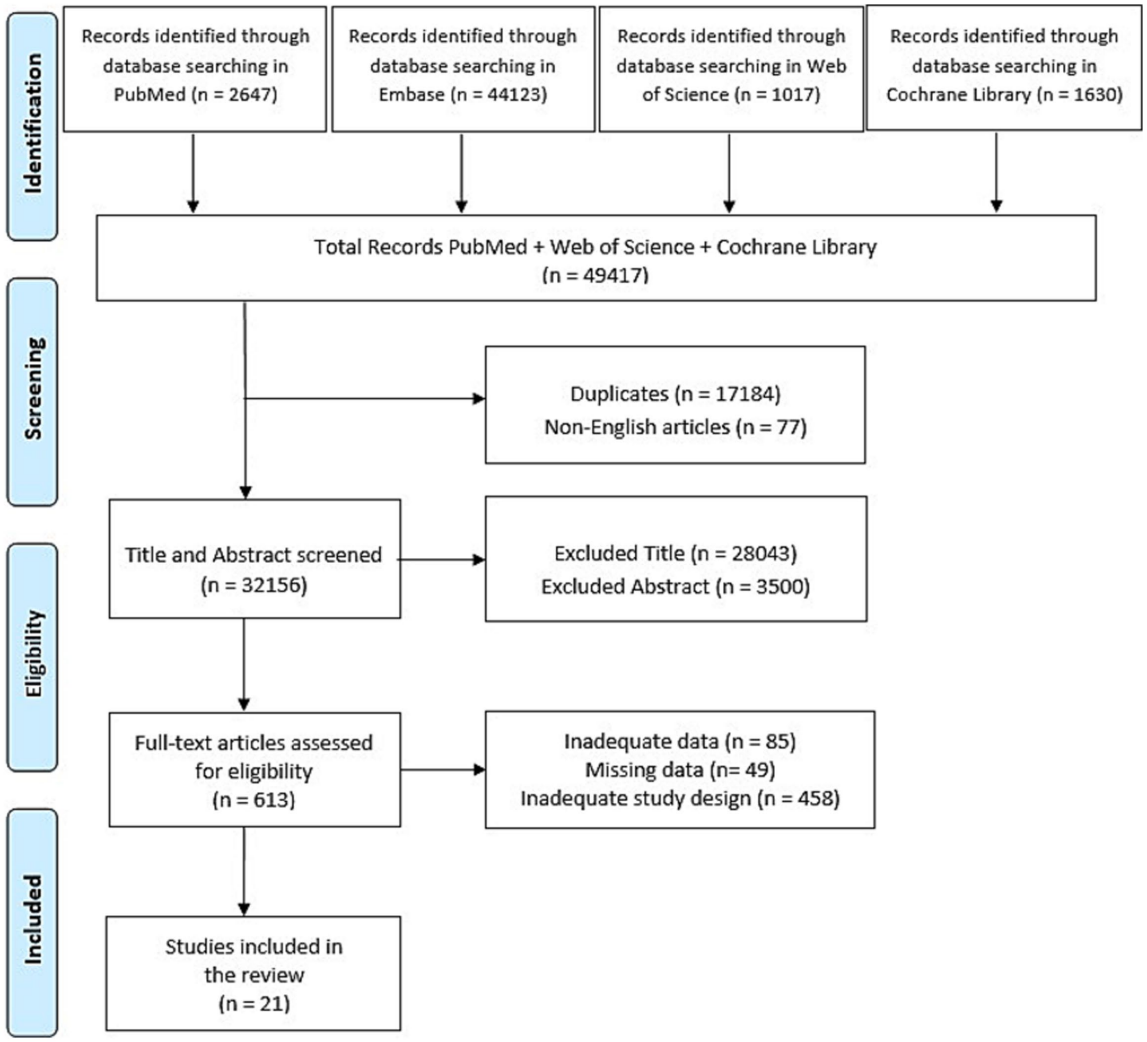
PRISMA flow chart reporting the study selection process.

The entire search procedure was reported in Figure 1.

### 3.1 Predictors

#### 3.1.1 Neurophysiological predictors

Venkataramani et al. (2023) extracted EEG and ECG features as well as clinical parameters. Frequency domain metrics such as the mean power spectral density (PSD) for Delta (0.5–4 Hz), Theta (4–8 Hz), Alpha (8–12 Hz), and Beta (12–30 Hz) bands, and the mean Burst Suppression Ratio (BSR) were derived from nine EEG channels. ECG features included mean and standard deviations across channels. The prediction task was framed as a binary classification (good or poor outcomes) with a dataset of 80 features (36 from EEG, 36 from ECG, and 8 from clinical data). EEG recordings were also used by Di Gregorio et al. (2022), four quantitative EEG (qEEG) features were extracted: z-score PSD, dominant frequency peak, permutation entropy, and mean amplitude. These electrophysiological measures were analyzed to determine their ability to discriminate between brain injury etiologies and to predict clinical outcomes at 6 months post-injury, distinguishing between patients who showed improvement and those who did not. Other authors, like Wang et al. (2022), included pain responses, arousal reactions, spontaneous movements, tendon reflexes, light reflexes, and vital signs within the predictors. These assessments were conducted in addition to EEG connectivity analysis. In the study by Tjepkema-Cloostermans et al. (2019), continuous EEG monitoring was used in addition to Cerebral Performance Category (CPC) scale in patients with post-cardiac arrest care. The authors included the combination of neurophysiological and clinical outcomes to compare the accuracy and reliability of automated DNN-based EEG analysis with traditional visual EEG assessments performed by experienced clinical neurophysiologists. This combined approach, between neurophysiological and clinical outcomes, was also implemented by Aellen et al. Both authors assessed the primary outcome of survival and awakening from coma 3 months post-cardiac arrest, CPC scale to classify survivors (CPC 1–3) and non-survivors (CPC 5). In addition, auditory event-related potentials (ERPs) were included in the dataset. ERPs were also used by Armanfard et al. (2019) with the primary outcome measure set to the detection of Mismatch Negativity (MMN), an ERP indicating auditory discrimination.

Lee et al. (2022), developed an Explainable Consciousness Indicator (ECI) using TMS-EEG and resting-state EEG, capable of distinguishing various states of arousal and awareness across sleep, anesthesia, and brain injury. ECI outperformed existing metrics like perturbational complexity index in differentiating conditions such as REM vs. deep sleep and ketamine vs. propofol-induced states and was effective in distinguishing MCS from UWS patients.

#### 3.1.2 Neuroimaging predictors

Campbell et al. (2020) analyzed three distinct datasets: one involving participants transitioning from wakefulness to general anesthesia under propofol, monitored with the Ramsay scale; another with sedated subjects classified by the Observer’s Assessment of Alertness/Sedation score; and a third comprising DoC patients evaluated with the CRS-R and undergoing fMRI to explore brain connectivity. fMRI scans were also used by Yang et al. (2024), using 3 different Tesla MRI systems (Philips Ingenia, GE Signa, and Siemens), and both functional and high-resolution anatomical images were acquired to aid in the localization of brain activity. Participants were instructed to rest with eyes closed in a wakeful, non-task state. As other authors, Zheng et al. (2017), investigated thalamo-cortical structural connectivity in DoC patients using diffusion tractography. Lastly, Liu et al. (2023), employed CT scans to predict consciousness levels based on structural brain damage.

#### 3.1.3 Autonomic predictors

Riganello et al. (2010), investigated heart rate variability (HRV) changes in response to emotional music stimuli. In healthy controls, self-reported emotional responses were used to classify HRV patterns associated with emotional valence. In UWS patients, HRV was used to evaluate whether emotionally charged auditory stimuli could elicit residual autonomic reactivity. Findings indicated that even UWS patients may exhibit autonomic responses to emotional stimuli, suggesting preserved emotional processing.

In the study of Wielek et al. (2018), the primary outcome measure was the identification of distinct sleep stages in DoC patients using ML-based classification. The supervised classification model was trained on healthy subjects’ polysomnography data, with traditional sleep staging based on the American Academy of Sleep Medicine (AASM) criteria. The classification was validated using video assessments, which identified periods of prolonged eye opening or closure. In the supervised approach, 11 DoC patients (5 MCS and 6 UWS) showed highly accurate sleep classification with an F1-score of 0.87, indicating strong overlap between predicted sleep stages and observed eye closure. The unsupervised clustering approach revealed a more complex pattern of sleep–wake states in MCS patients. This suggests that the presence of structured sleep, whereas UWS patients showed no such clustering, indicating a highly fragmented sleep pattern.

#### 3.1.4 Clinical predictors

In the study of Magliacano et al. (2023), the primary outcome measure was the recovery of full consciousness, assessed using CRS-R scores. A novel Consciousness Domain Index (CDI), derived using unsupervised machine learning from CRS-R sub scores, was validated against clinical diagnoses and CRS-R total scores for prognostic accuracy. CDI showed superior sensitivity and specificity in predicting outcomes at 6, 12, and 24 months, outperforming traditional measures. The CDI classified patients into two clusters, revealing that motor, visual, and auditory CRS-R sub scores were the most critical features for outcome prediction. In a similar way, Campagnini et al. (2024), used CSR-R-derived metrics including total and subscale scores, CRS+ (weighted scores), and the CDI for their predictive utility. Narayanan et al. (2023) used two noninvasive neurobehavioral assessment tools: the Music Therapy Assessment Tool for Awareness in Disorders of Consciousness (MATADOC) and the Coma Recovery Scale-Revised (CRS-R) to predict prolonged DoC diagnostic states. In another study, Zheng et al. (2022) assessed neurological outcomes at three-or six-months post-arrest using the CPC scale, categorizing outcomes as good (CPC 1–2) or poor (CPC 3–5). Poor outcomes were observed in 64% of the cohort. Liuzzi et al. (2022) included patients’ demographical and medical data in addition to clinical scales to measure level of consciousness (e.g., CRS-R), functional disability (e.g., Disability rating scale, DRS), and level of clinical complexity (e.g., Early Rehabilitation Barthel Index, ERBI), medical comorbidities before the brain injury (e.g., Cumulative Illness Rating Scale, CIRS), and presence of medical devices (e.g., for supporting respiratory functions, feeding).

In a study of El-Rashidy et al. (2023) specific patients’ data, through three phases: vital sign acquisition, Fog-Assisted Consciousness Management (FACM), and cloud computing integration. Vital sign data were collected through IoT sensor devices, wearable monitors, and medical reports. The FACM phase managed data transmission between local devices and cloud servers, optimizing processing and storage. The cloud infrastructure facilitated large-scale data analysis and clinical service delivery, aiming to transition healthcare data management from conventional storage to a fully digitalized framework.

In the study of Molteni et al. (2019), prognostic assessments were conducted at two time points: 3 months (T0) and 6 months (T1) after hospital admission, using the Levels of Cognitive Functioning (LCF) assessment scale. Prognostic outcomes were categorized into four levels 5 years after admission: death (0), UWS (1), MCS (2), and emergence from MCS (exit MCS = 3). The study aimed to develop a predictive model that could classify patients into these categories while identifying the most relevant neurobehavioral assessment domains contributing to prognosis. In another study by Liuzzi et al. (2022), the authors investigated the impact of medical complications (MCs) on the prediction of clinical outcomes in patients with prolonged disorders of consciousness (pDoC) using machine learning models. The primary goal was to determine whether integrating MC at 3 months after the event could enhance the predictive accuracy of functional outcomes at 6 months post-injury, assessed using the Glasgow Outcome Scale-Extended (GOS-E).

Unlike other studies, Muller et al. (2019) used serial NSE measurements over 18 days post-ICU admission to predict 1-year neurological outcomes via CPC. Neurological outcomes were assessed at 1 year using the Clinical Performance Category scale, classifying patients into five outcome groups: dead, vegetative state, severe disability, moderate disability, and good recovery.

### 3.2 AI analysis approaches in DoC studies

We found that the selected evidence applied various AI-based approaches for both diagnostic and prognostic purposes. In this result’s section, we divided into two parts based on the evidence’s aim.

#### 3.2.1 Diagnostic approaches

DL techniques were implemented by Lee et al. (2022), in which the analysis approach relied on a CNN trained on spatiotemporal EEG features extracted from TMS-EEG responses. The model was optimized using a leave-one-subject-out (LOSO) cross-validation strategy and applied domain transfer learning to improve generalization across sleep, anesthesia, and brain injury datasets. Layer-wise relevance propagation (LRP) was employed to interpret the CNN’s decision-making process, revealing that EEG activity in the parietal cortex played a key role in distinguishing consciousness states. Similarly, to distinguish between conscious and unconscious states using rs-fMRI-derived features, Campbell et al., compared three ML models: support vector machine (SVM), Extra Trees (ET), and Artificial Neural Networks (ANN). These models were trained on features extracted from fMRI scans of participants under anesthesia and DoC patients, with the goal of identifying biomarkers that could be generalized to clinical populations. SVM, ET, and ANN models all showed varying degrees of success in differentiating levels of consciousness, with ANN exhibiting strong performance in detecting non-linear patterns in brain connectivity data.

Di Gregorio et al. (2022) used linear discriminant analysis (LDA) with LOSO cross-validation to classify patients into improved vs. non-improved groups based on EEG features. The classification was performed separately for TBI and non-TBI patients, allowing for a tailored evaluation of EEG biomarkers within each etiology. Given the retrospective nature of their study, the analysis accounted for class imbalance by incorporating balanced accuracy and precision metrics. Only EEG variables that demonstrated significant group differences in the initial analyses were included in the LDA model to maximize classification performance.

Wielek et al. (2018) used combined supervised and unsupervised ML methods to classify sleep stages based on EEG-derived permutation entropy features, a measure of signal complexity. The supervised classification used RF and feedforward neural networks trained on healthy sleep data and tested on DoC patients. The unsupervised approach employed hierarchical clustering to analyze group-level sleep patterns, revealing that MCS patients exhibited sleep organization more similar to healthy individuals, while UWS patients lacked structured sleep cycles.

In a similar way, Yang et al. (2024) tested the classification performance of DeepDOC in distinguishing DoC patients from healthy controls and, subsequently, in differentiating MCS from UWS patients. DeepDOC was compared to five state-of-the-art ML models: linear SVM, logistic regression (LR), random forest (RF), extreme gradient boosting (XGBoost), and adaptive boosting (AdaBoost). Performance was evaluated using accuracy and area under the curve (AUC) metrics. DeepDOC achieved superior results, with an AUC of 0.927 and an accuracy of 0.861 in distinguishing MCS from UWS patients. Additionally, the framework excelled in identifying covert motor dysfunction, achieving an AUC of 1 and an accuracy of 0.909. The study also utilized gradient-weighted class activation mapping (Grad-CAM) to interpret the model’s decision-making, revealing that the posterior cortex, including the visual cortex, played a key role in distinguishing consciousness states.

Narayanan et al. (2023) evaluated the accuracy of ML models in classifying prolonged DoC diagnostic states using supervised and unsupervised learning techniques. Supervised learning methods included decision trees as well as ANNs, both trained using computed diagnostic results to achieve optimal classification. Cross-validation using a 10-fold strategy ensured robust model evaluation, and diagnostic accuracy was assessed through the consistency and predictive reliability of the ML models. Network analysis was also applied to explore potential transitions between prolonged DoC states, aiming to provide novel insights into patient progression. The analysis approach implemented by the authors was useful to effectively classify DoC states using only 13 key variables, eliminating the need for extensive clinical or imaging datasets. Furthermore, the authors also applied an unsupervised based approach (i.e., k-means) to identify hidden patterns in patient data, particularly in cases where there was low agreement in computed diagnostic outcomes. Decision trees and ANNs achieved high predictive accuracy, while k-means clustering revealed distinct patient subgroups with shared diagnostic characteristics.

Riganello et al. (2010), used data mining procedures were applied to identify significant changes in HRV, which is considered as a biomarker of autonomic correlate of brain activation, in response to complex auditory stimuli associated with emotional value (i.e., music). The data mining techniques were used to derive significant patterns and associations from the dataset. The open-source software WEKA was employed to train decision trees and identify association rules. A 1-R rule-based classification system was developed, where reported emotions were treated as target variables, and HRV parameters served as predictors. This approach successfully modeled emotional states based on HRV data and revealed that autonomic changes with potential emotional valence could be induced in VS patients, offering new insights into their residual reactivity and the broader implications for emotional processing in disorders of consciousness.

In the study by Wang et al. (2022), a novel EEG connectivity measure, the PSD difference incorporating a recursive cosine function (CPSDD), was introduced to enhance classification performance. The Ensemble-Of-SVM classifier consisted of 100 individual SVM models, each trained to classify patients as DoC (+) or awake (−). The final prediction was obtained through a majority-voting scheme across all models. The classifier’s performance was benchmarked against 12 alternative connectivity measures. Among these, the EOSVM using the CPSDD feature yielded the best results, with an accuracy of 98.21, 100% sensitivity, and 95.79% specificity.

Similarly, Zheng et al. (2017), assessed the classification accuracy of structural connectivity patterns in differentiating UWS, MCS−, and MCS+ patients. Probabilistic diffusion tractography was performed to estimate probability distribution functions for fiber directions in each voxel. Two main analyses were conducted: quantifying thalamic connectivity to cortical targets and generating path distribution maps to visualize these projections. These outputs were compared across patient groups to evaluate how thalamo-cortical connectivity varied between UWS, MCS−, and MCS+ patients. A multivariate classification approach using cross-validated SVM and reflector mapping identified the most discriminative regions along thalamo-cortical tracts. They used a reflector mapping technique combined with an e-2-subjects-out cross-validated SVM was used to identify the most discriminative regions along the thalamic tracts. Voxel-wise accuracy maps were computed and tested for statistical significance using inverse binomial distribution. Results showed that MCS+ patients exhibited more preserved thalamo-cortical connectivity than MCS− and UWS patients.

#### 3.2.2 Prognostic approaches

Different authors implemented DL approaches for prognostic purposes. For example, in the study by Tjepkema-Cloostermans et al. (2019), convolutional neural networks (CNNs) were trained and validated using five-minute EEG epochs recorded 12- and 24-h after a cardiac arrest. EEG signals were preprocessed with filtering, subsampling, and re-referencing via bipolar and Laplacian montages. Binary cross-entropy served as the loss function, and the network’s output was the probability of a good neurological outcome, averaging 30 EEG fragments per five-minute epoch. Separate networks were trained for each montage and time point, and the prognostic value of EEG temporal evolution was also analyzed. The study predicted good neurological outcomes with 48% sensitivity at a 5% false positive rate, and poor outcomes with 58% sensitivity and 0% false positives at 12 h post-arrest. The implementation of deep neural networks (DNNs) allowed for more accurate prognostication in a larger proportion of patients, particularly at early post-cardiac arrest time points. This approach demonstrated the potential of temporal EEG and DL to enhance individualized outcome prediction and support ICU decision-making. Aellen et al. (2023) employed EEGNet, a CNN, to predict outcomes in comatose patients. A 10-fold cross-validation method divided patients into training (60%), validation (20%), and test (20%) sets. The model used binary cross-entropy loss, Adam optimizer, and early stopping for training. Confidence scores for survival were derived from averaged predictions across trials. Predictions were based on averaged confidence scores across single-trial EEG classifications. Performance metrics included the AUC mean of 0.70 ± 0.04 on the test set, with a positive predictive value (PPV) of 0.83 ± 0.03 and a negative predictive value (NPV) of 0.57 ± 0.04. Additionally, for patients with indeterminate clinical prognoses (“gray zone”), the CNN achieved a PPV of 0.86 and an AUC of 0.75, underscoring its utility in uncertain cases. Neural synchrony (phase-locking value) and complexity (Lempel-Ziv) correlated with CNN confidence, with higher synchrony and lower complexity indicating survival. This approach demonstrated strong interpretability and robust predictions, even for patients in the clinical “gray zone.” In the study by Zheng et al. (2022), a DL model exploiting bidirectional long-short term memory (Bi-LSTM) networks was developed to analyze EEG dynamics over time. The model utilized clinically interpretable EEG features such as burst suppression ratio, band power metrics, and spike frequency, derived from five-minute epochs. Predictions of poor neurological outcomes were made by analyzing temporal trends, with the model improving accuracy as additional EEG data accumulated over 66 h. Sensitivity and specificity were calculated at various thresholds, and model performance was measured by the area under the receiver operating characteristic curve (AuC-ROC). The Bi-LSTM model significantly outperformed state-of-the-art approaches such as convolutional neural networks and random forests. It achieved a peak AuC-ROC of 0.88 at 66 h, demonstrating the value of incorporating long-term EEG trends. Calibration curves confirmed high concordance between predicted and actual outcomes.

In the study of Venkataramani et al. (2023), XGBoost was used for prognostic prediction due to its ability to handle small datasets with missing values, with hyperparameters fine-tuned for optimal performance. A random forest (RF) regressor was employed for CPC prediction. Validation was performed using 5-fold stratified cross-validation to ensure balanced outcome representation, achieving a cross-validation score of 0.34 and an official challenge score of 0.381. While convolutional architectures like AlexNet and ResNet were tested, they were less effective due to high false positive rates despite their high accuracy.

Most of the studies employed supervised machine learning techniques for outcome prediction, while some, like Magliacano et al. (2023) combined unsupervised and supervised approaches to derive and validate prognostic indices. In particular, Campagnini et al. (2024), applied supervised ML models, such as LR, SVM, RF, and k-nearest neighbors (KNN) algorithms. Nested cross-validation ensured robust model evaluation, with missing data imputed using KNN-based methods. Performance was validated using F1-scores and AIC/BIC values across folds. These authors suggested that CRS-R subscale scores, particularly when integrated into machine learning frameworks, offer superior prognostic value, enabling personalized rehabilitation plans with minimal reliance on complex diagnostics. Magliacano et al. (2023) employed k-means clustering with cross-validation to derive the CDI from CRS-R subscores and applied LR to evaluate its association with outcomes. Confounding factors such as age, sex, and injury type were included in multivariate analyses. The CDI improved the prognostic accuracy of recovery predictions over conventional measures, particularly for younger patients, those with TBIs, and those assessed early post-injury. The study supports the use of ML-enhanced indices like CDI for personalized prognosis in pDoC, especially in younger or TBI patients assessed early post-injury. In the study by Armanfard et al. (2019), the Localized Feature Selection method was employed to optimize feature extraction from EEG signals. Unlike traditional feature selection methods, this method assigns a unique feature set to each training sample, enabling the model to handle non-stationarity, disjoint class clusters, and non-linear decision boundaries. This method was particularly effective in addressing challenges of small sample sizes and high-dimensional datasets, reducing overfitting risks. The machine learning framework achieved an impressive accuracy of 92.7% on the healthy training set, as evaluated using a Leave-One-Subject-Out (LOSO) cross-validation procedure. Liu et al. (2023) in their study integrate statistical modeling with AI-based image analysis in the analysis approach. Hemorrhage volume, related to brain hemorrhage, was automatically calculated using the DEEPWISE Medical AI software, ensuring accuracy and eliminating human errors in volume estimation. LR modeling confirmed that hemorrhage volume and ventricular involvement were the most reliable indicators of consciousness status, outperforming other CT-derived features such as hematoma shape, density, and midbrain involvement. Liuzzi et al. (2022), trained and tested four ML models: Elastic-Net (EN), Orthogonal-Matching Pursuit (OMP), KNN, and Support Vector Regressor (SVR). A five-fold cross-validation strategy was used, ensuring robust performance evaluation across different patient subsets. To address dataset imbalance, the Synthetic Minority Oversampling Technique (SMOTE) was applied to resample the training sets. Hyperparameter optimization was performed to minimize cross-validation error, with the models’ performance evaluated based on prediction accuracy. Initial results showed an 88.6% accuracy in predicting functional outcomes at admission, which improved to 92.6% when MC data were incorporated into the model. The analysis demonstrated that accessible clinical features, combined with relatively simple ML algorithms, could achieve high accuracy in predicting pDoC patient outcomes without the need for specialized instrumental assessments.

## 4 Discussion

In this scoping review, we explored the use of AI, including ML and DL, in the context of DoCs. The selected studies highlighted different methodologies and parameters for assessing and predicting outcomes in patients with DoC. Several parameters ranged from advanced EEG analyses, neuroimaging to behavioral assessments, analyzed within AI (in particular ML and DL) applications. For example, Venkataramani et al. (2023) combined EEG and ECG data to predict post-anoxic coma recovery using tree-based ML algorithms, while Aellen et al. (2023) used CNNs to extract EEG features predictive of coma awakening and long-term survival. In addition, they also used CNNs to extract single-trial information from auditory ERPs on the first day of coma, and at predicting survival 3 months later. In line with our results, the literature review by Lee and Laureys (2024) revealed that most of the evidence in this field is focused on AI for diagnostic purposes, differentiating between UWS and MCS. Another aspect is related to the use of clinical scales like GCS or LCF to predict recovery. While these scales are widely accepted, they may not fully capture the complexity of DoC recovery, compared to the multidimensional assessment done via the CRS-R. Furthermore, the possibility to integrate instrumental data, including behavioral, genetic, and neurophysiological, neuroimaging markers has shown the potential to improve outcome prediction.

### 4.1 Key parameters: what is the role of AI in the diagnosis and rehabilitation of DoCs?

Based on the selected evidence, certain neurophysiological parameters appear to be crucial for extraction, as they provide valuable insights for prognostic assessments. For instance, Venkataramani et al. (2023) used EEG-derivated bands (i.e., delta, theta, alpha, and beta bands) along with the Burst Suppression Ratio (BSR), extracted from EEG, to assess post-anoxic coma recovery. When BRS is high, it indicates a greater proportion of suppression periods, which can be associated with deeper levels of unconsciousness or impaired brain function. These results are in line with those found by Forgacs et al., in terms of corticothalamic and corticocortical activity, measured in resting state EEG (Forgacs et al., 2017).

Similarly, ERPs are often derived from EEG data, as in Aellen et al. (2023), to evaluate sensory responses to stimuli and predict survival rates. While Armanfard et al. (2019) found that MMN reflects automatic cortical responses to auditory stimuli and is a reliable indicator of cortical reactivity. The MMN is a negative-going ERP waveform typically observed when there is a deviation or “mismatch” between a regularly occurring stimulus and a rare, unexpected one (the deviant stimulus). Brain’s automatic detection of this mismatch or change in the environment seems to be reflected by the MMN response even when the individual is not consciously attending to the stimulus.

According to the selected evidence in this review, EEG-based measures, especially qEEG and functional connectivity, are valuable tools for assessing rehabilitation potential and guiding clinical decision-making. Furthermore, the results Di Gregorio et al. (2022), suggested that combining multiple EEG biomarkers using ML techniques enhances predictive accuracy, reinforcing the role of EEG as a critical tool in DoC prognosis and patient management.

Crucially, AI was employed to extract information from neuroimaging data, as in Zheng et al. (2017) where the capability of diffusion tractography to assess structural connectivity, at varying levels of consciousness, was assessed. Yang et al. (2024) used rs-fMRI to measure the connectivity between brain regions, offering a deep understanding of altered states of consciousness, identifying subtle neural patterns indicative of awareness in patients with DoC. The findings by Campbell et al. (2020) suggested that ML classifiers trained on rs-fMRI data from anesthetized subjects may serve as a valuable tool for identifying neural signatures of unconsciousness, potentially improving diagnostic accuracy in clinical settings. This study supports the feasibility of using ML to refine DoC assessment and underscores the potential of anesthesia-based training data in developing robust predictive models for impaired consciousness.

In addition, parameters such as hemorrhage volume and ventricular involvement were extracted, as seen in Liu et al. (2023), for assessing outcomes in patients with brainstem hemorrhages. These findings suggested that CT data could be predictive of consciousness recovery or progression in brainstem hemorrhage patients (Liu et al., 2023). In this way, clinicians can exploit a rapid method for assessing consciousness status, aiding in early diagnosis, treatment decisions, and surgical planning for DoC patients.

In another study, Riganello et al. (2010) found that HRV serves as a marker of autonomic reactivity and emotional processing, even in patients with minimal consciousness. According to some authors, this autonomic parameter is fundamental to monitor potential recovery of consciousness. In addition, it can be considered as a non-invasive, easy, and low-cost way to monitor patients’ vital status. Similarly, Wielek et al. (2018), with polysomnography recordings and sleep patterns, demonstrated that ML can improve sleep staging in DoC patients, offering an objective and automated method to assess residual brain functioning and potentially contributing to prognosis and patient management. In this sense, the role of AI can improve the use of autonomic and vital functions in current clinical practice, suggesting their role for diagnostic purposes.

Despite the promising results across these studies, a limitation lies in the variability of the parameters selected for analysis through ML and DL techniques. In this review, we reported the key features highlighted by the authors; however, there is still no consensus on which of the reported parameters are the most robust or reliable for diagnosis and/or prognosis in DoC. This lack of standardization may limit comparability across studies and the clinical implementation of these tools. Another limitation concerns the limited availability of advanced neuroimaging technologies, such as fMRI, which are not accessible in all clinical centers. This may restrict the widespread implementation of AI models that rely on such high-resolution data, especially in low-resource settings, and highlights the need for using accessible diagnostic tools.

### 4.2 Type of analysis: what are the most used AI technologies in the field of DoCs?

The studies employed various analysis techniques including both ML and DL approaches, as well as traditional statistical methods. It is noteworthy that the choice of technique depends on the data type, volume, and clinical objectives. According to our results, DL techniques seem to be ideal for complex, high-dimensional data (e.g., rs-fMRI), offering superior predictive power but with higher cost of interpretability and computational demands. On the other hand, traditional ML models like RF and SVM are preferred for smaller datasets with clear feature sets, balancing performance and interpretability. Lastly, Hybrid Approaches that combine ML with IoT infrastructure are promising for dynamic, real-time assessments, particularly in clinical environments with continuous monitoring needs (Ala et al., 2024).

#### 4.2.1 Supervised ML techniques

Supervised ML techniques were used by several authors like Wang et al. (2022) and Molteni et al. (2019). In particular, Wang et al. (2022) used SVM for binary classification tasks (e.g., DoC vs. awake states). They developed an ensemble of multivariate SVMs (EOSVM) to classify patients into DoC (+) or awake (−) states based on EEG-derived connectivity measures, including their novel CPSDD metric. Notably, the 97% majority voting in EOSVM significantly enhances reliability. Notably, 35% of patients were diagnosed with an accuracy of 98.21%, a sensitivity of 100%, and a specificity of 95.79%. In a similar way, Zheng et al. (2017) applied binary SVM classifiers to thalamo-cortical connectivity features extracted via probabilistic tractography to distinguish between MCS and VS patients.

Using SVM in clinical context can be effective to analyze high-dimensional data like EEG features and for binary classification tasks with limited training samples (Zheng et al., 2017; Wang et al., 2022), one of the main struggles of the research in the neurorehabilitation field (Morone et al., 2024).

Other supervised learning techniques include RF that was implemented by Molteni et al. (2019). In particular, Molteni et al. (2019) applied RF for multi-class prediction in pediatric DoC patients. The model was used to classify recovery levels based on neurobehavioral data, and feature important scores helped interpret predictions. In this case, the advantage of using RF lies in the possibility to hold missing data and unbalanced datasets effectively. In addition, it provides interpretable results through feature importance rankings. However, RF can show limitations with large datasets with many trees, and it can be sensitive to noisy features.

Liuzzi et al. (2022) used another ML technique such as Elastic-Net regularized LR for predicting functional outcomes in DoC patients, combining clinical observations and vital signs. The pros of using this type of analysis can be related to the fact that it can be applied to small datasets, however the main cons of LR are that it assumes linear relationships, which might not capture complex interactions.

#### 4.2.2 Deep learning techniques

CNNs were used by Aellen et al. (2023) to extract features from EEG responses to auditory stimuli for survival prediction at 3 months post-cardiac arrest. While Yang et al. (2024) introduced a 3D EfficientNet-B3-based CNN (DeepDOC) to classify rs-fMRI data into MCS and UWS categories. This approach used two sequential networks to identify DoC patients and further differentiate between subgroups. The CNNs have a great advantage in managing high-dimensional, unstructured data like EEG or fMRI images. However, they require large datasets for training, which are often unavailable in clinical research.

On the other hand, recurrent neural networks (RNNs) such as Bi-LSTM were used by Zheng et al. (2022) to analyze temporal EEG trends. The model captured temporal dependencies to predict neurological outcomes, with accuracy improving as more data was included. This approach is ideal or temporal and sequential data like EEG trends.

### 4.3 Clinical implications: how can AI improve patients’ care?

**Figure 2:**
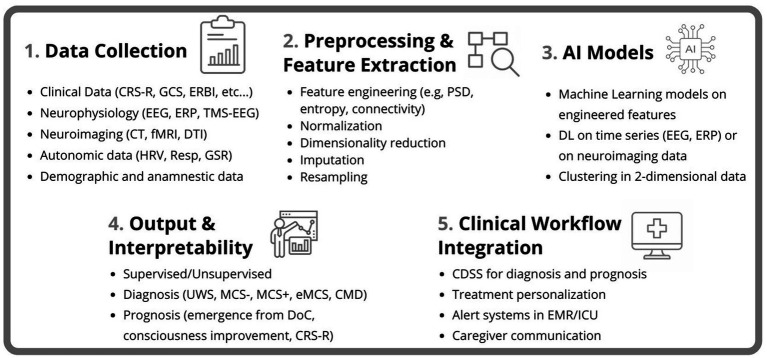
Overview of the AI-based pipeline for DoC assessment. The workflow includes five main stages: (1) Data Collection from clinical, neurophysiological, neuroimaging, autonomic, and demographic sources. (2) Preprocessing and Feature Extraction involving signal processing, normalization, imputation, and dimensionality reduction. (3) Application of AI Models, including machine learning and deep learning approaches. (4) Output and Interpretability for diagnostic and prognostic purposes; and (5) Clinical Workflow Integration, supporting decision-making, treatment personalization, and communication with caregivers.

In this scoping review, we have also analyzed the advantages and disadvantages of using AI technologies in the field of DoCs, suggesting potential clinical implications that could be applied in future studies. As a strength, AI methods allow multimodal integration, combining neurophysiological, behavioral, and imaging data enhancing diagnostic precision and prognostic accuracy. In addition, this multimodal integration of clinical data of the patient is fundamental to achieve an overall vision of the medical condition and a patient-centered care approach (see Figure 2).

Diagnosis of DoCs is a complex challenge, deriving from the fact that DoC is a neurological condition caused by a disease, such as brain hemorrhage, post-anoxic state, tumors and/or TBI. Moreover, there are behavioral differences among various clinical presentations of DoC. This extreme heterogeneity in terms of etiology and clinical presentation makes the diagnostic classification of DoC even more complex. In this context, AI could assist clinicians by providing insights that help them better diagnose patients with DoC. Some authors (Di Gregorio et al., 2022; Lee et al., 2022; Zheng et al., 2022; Campagnini et al., 2024; Yang et al., 2024) have incorporated results related to the interpretability of AI algorithms performance in their studies. This allows clinicians to better understand why the AI made a particular prediction at the patient-level, facilitating its potential integration into clinical practice, extending possibilities for treatment personalization. However, few studies have investigated the aspect related to the interpretability of AI algorithms, thus future studies could include data on this important aspect.

In terms of prognosis, the recovery of consciousness depends on various factors related to basic physiology, such as cardiovascular and respiratory functions, as well as secondary aspects like new brain hemorrhages, internal medicine complications, and infections. These factors can influence DoC patients’ recovery, which, as mentioned before, is not a single disease but it is a multi-domain condition, since it impacts the whole body’s physiology. In this regard Estraneo et al. (2018) found, through separate binary multivariable LR analyses, that patients with male sex and endocrine-metabolic complications in MCS patients were independent risk factors for mortality at all stages. Furthermore, older age, anoxic etiology, lower CRS-R scores, and a VS diagnosis at study entry were linked to a lower chance of clinical or functional improvement in survivors. In survivors, epilepsy was associated with no clinical improvement only at 24 months post-onset. Similarly, Liuzzi et al. (2022) developed and validated an interpretable decision support tool capable of identifying patients who will achieve a sufficient level of independence (GOS-E > 4) within 6 months. Additionally, at 3 months, the model provided an updated prediction, considering the rehabilitation process and newly emerged medical complexities. In this sense, an AI-based approach can be used to tailor patients’ treatment and rehabilitation path, thanks to the continuous recording of neurophysiological parameters (e.g., neuroimaging, HRV) and clinical assessment (e.g., CRS-R). This aspect is also important when the patient needs to start the intervention, as early as possible. In fact, prognostic models could identify timely identification of patients with higher recovery potential, ensuring efficient resource allocation. On the other hand, we sought to explore the weaknesses of AI technologies in the field of DoCs. Firstly, many studies rely on limited homogenous samples. However, the etiology of DoCs can vary from traumatic to acquired brain injury and it can affect people of all ages. This is why it is difficult to obtain a homogenous sample of patients to increase generalizability. Another weakness is related to the differences in data acquisition (e.g., EEG configurations, neuroimaging protocols) among the selected studies. Lastly, it is noteworthy that while advanced ML models excel in accuracy, they often lack clinical interpretability, which is essential for decision-making. This is why multidisciplinary collaboration between biomedical engineers, computer scientists and clinicians, is necessary in order to obtain a technologically advanced, easy-to-use and low-cost tool that can aid to solve problems in the clinical field. Furthermore, future developments of AI in DoC must consider ethical aspects, which play a crucial role, particularly regarding the use of neural data for training algorithms. Neuroethics demands careful evaluation of patient consent, data privacy, and the potential biases embedded in AI models (Ienca and Ignatiadis, 2020; Ienca, 2021). In the case of DoC patients, consent for data acquisition cannot be given directly but must be provided by their legal guardian, raising ethical concerns about autonomy and representation. Ensuring transparency, accountability, and fairness in AI-driven DoC diagnostics and rehabilitation will be essential to increase trust and maximize the benefits of these technologies while safeguarding patient rights.

It is important to acknowledge the limitations of the reviewed studies, and the scoping review approach employed. First, the generalizability of many findings is constrained by small sample sizes (Riganello et al., 2010; Wielek et al., 2018; Armanfard et al., 2019; Lee et al., 2022). Secondly, many studies (Di Gregorio et al., 2022; Liuzzi et al., 2022; Liu et al., 2023; Yang et al., 2024) are often based on retrospective data and would benefit from prospective validation to support their translation into routine clinical practice. Regarding the limitations of our scoping review, the exclusion of non-English papers may have resulted in the omission of relevant studies, and the lack of statistical analysis limits the quantitative assessment of the evidence. As a result, our scoping review provided a comprehensive qualitative synthesis of the available evidence, offering valuable insights into the role of AI technologies in the field of diagnosis and prognosis of DoC patients, identifying key implications for clinical practice and considerations for future investigation.

## 5 Conclusion

Advancing AI applications in DoC requires several key developments such as establishing standardized protocols for data acquisition and preprocessing, considering variations in demographic data and underlying etiologies. Finally, the potential integration of AI technologies into clinical management of DoC could be helpful in diagnosis and prognosis. Diagnosing DoC is inherently complex, but exploiting clinical, neurophysiological, laboratory, and neuroimaging data, could provide a more comprehensive understanding of each patient, enhancing the reliability of AI algorithms. Distinguishing among the different alterations of consciousness could be a challenge for the clinicians. AI-based technologies could support this aspect, facilitating the distinction between the different states of DoC, such as UWS and MCS. Furthermore, AI can contribute to improve and personalize rehabilitative treatment by identifying key factors that influence recovery and incorporating them into rehabilitation protocols, ultimately optimizing patient outcomes.
