# Supplementary material for: Can artificial intelligence improve the diagnosis and prognosis of disorders of consciousness? A scoping review
Source: Front Artif Intell. 2025 May 30;8:1608778. doi: 10.3389/frai.2025.1608778 (PMC12162576; doi:10.3389/frai.2025.1608778)
Supplement: Supplementary file 2 [file Table_1.pdf]

Table 1. Summary of the included studies.

Reference	Aim	Sample size	Type of intervention	Predictors	Type of analysis	Model performance
(Venkataramani et al., 2023)	Creation of a ML-based solution for coma prognosis after cardiac arrest using longitudinal EEG and ECG recordings for the<br><br>Predicting Neurological Recovery from Coma After Cardiac Arrest.	607 patients in post-anoxic coma	Intervention category: Prognosis<br><br>Characteristics of the intervention: In this study, it was decided to use only EEG data and EEG data from the provided signal data and all the features of the clinical data. In addition, both EEG and EEG data were preprocessed, including filtering, resampling, smoothing and scaling steps before featurization.	Age, sex, a hospital identifier, the arrest location, and the type of cardiac rhythm recorded during resuscitation and signal data, including EEG (Delta, Theta, Alpha and Beta) and mean Burst Suppression Ratio, ECG.	.<br>DT, RF, and GBT each offered distinct strengths. However, XGBoost emerged as the top choice due to its versatility and cutting-edge performance on various tasks boasting scalability, flexibility, a missing data handling capability, and custom loss functions.	The proposed solution achieved a challenge score of 0.381 on the hidden test set. While the score is modest, the model has a very low false positive rate, making it a useful tool for helping doctors identify patients for more targeted, in-depth evaluation.
(Armanfard et al., 2019)	This study proposes a machine learning-based framework that can accurately detect the presence of MMN, thus offering the possibility of improving the sensitivity of coma emergence prediction beyond the current value of 30% and thus improving the accuracy of the emergency test.	22 healthy subjects and 2 coma patients.	Intervention category: Prognosis.<br><br>Characteristics of the intervention: In this study, N1 (mandatory sensory response) and MMN (automatic response to auditory stimuli) components were evoked<br>using a modification of a classical auditory oddball paradigm. Stimuli consisted of standard tones (85%) and deviant tones (15%). These stimuli were presented randomly. All healthy subjects and comatose patients were exposed to the passive oddball paradigm consisting of standard and deviant tones. EEG was instead recorded with a 32-channel BioSemi headset. The recording incorporated an analog band-pass filtering operation that was digitally filtered offline.	MMN ERP	The LFS method was selected for machine learning as it assigns a unique feature set to each training sample, enabling the model to handle non-stationarity, class heterogeneity, and non-linear boundaries, while also reducing overfitting and maintaining strong performance in high-dimensional, low-sample-size settings..	The proposed machine learning method, evaluated using a Leave-One-Subject-Out (LOSO) procedure on the healthy training set with an accuracy of 92.7%, enables a significant reduction in the time window used for averaging ERPs. This shorter window minimizes the smearing effect caused by latency jitter and reveals waxing and waning cycles in the state of consciousness of comatose patients.
(Aellen et al., 2023)	Verify if CNNs would be able to extract patterns of EEG responses to standardized auditory stimuli that relate to patients' chances of awakening from coma and survival at 3 months.	145 comatose patients following cardiac arrest.	Intervention category: Prognosis.<br>Characteristics of the intervention: Patients were presented with a series of pure tones. Standard sounds were presented in 70% of the trials and had a pitch of 1000 Hz and duration of 100 ms. Deviant sounds differed from the standards in duration (150 ms), interaural time difference, with left ear leading with 700 μs, or pitch at 1200 Hz.	EEG, Glasgow Coma Scale (GCS), Cerebral Performance Category (CPC)	To predict patient outcomes, a CNN was trained on EEG data using 10-fold cross-validation, ensuring each patient's data appeared in only one of the training, validation, or test sets. The network was moderately sized to allow faster training while maintaining strong performance.	It has been proven that CNNs are powerful in extracting single-trial information from auditory ERPs on the first day of coma, and at predicting survival 3 months later, with a positive predictive power of 0.83±0.03, negative predictive power of 0.57±0.04 and an overall AUC of 0.70±0.04.
(Tjepkema-Cloostermans et al., 2019)	To more reliably and accurately predict the neurological outcome	895 consecutive comatose patients after cardiac arrest.	Intervention category:<br>Prognosis.	Performance Category (CPC), EEG	A CNN was trained using 5-minute EEG epochs recorded at 12 and 24 hours after cardiac arrest. Binary cross-entropy was used as the loss	This study demonstrated that DNN can reliably predict the neurological outcome of patients in coma after cardiac arrest in

	of patients in a coma after cardiac arrest, using deep neural networks instead of visual EEG by experienced clinical neurophysiologists.		Characteristics of the intervention: Patients were treated according to standard protocols for patients in a comatose state after 6 months of cardiac arrest. Continuous EEG recordings were started as soon as possible after admission to the ICU and continued for up to 3 to 5 days, unless patients regained consciousness or died at an earlier stage.		function. The model output was the average probability of a good neurological outcome across 30 EEG fragments. Separate networks were trained for each montage and time point, and the temporal evolution of EEG was also analyzed for its prognostic value.	substantially more patients than visual EEG assessment. The predictive values were also higher than neurological examination or SSEP. Poor outcome was predicted with a sensitivity of 58% at a FPR of 0% at 12 hours after cardiac arrest. Good neurological outcome could be predicted at 12 hours after cardiac arrest with a sensitivity of 48% at a FPR of 5%.
(Magliacano et al., 2023)	The proposed objectives of the study included externally validating the prognostic accuracy of CDI relative to the patient's clinical diagnosis and CRS-R total score and investigating the predictive accuracy of CDI on long-term recovery of consciousness at 6, 12, and 24 months after injury.	143 patients with prolonged DoC , of which 75 in MCS and 68 UWS.	Intervention category: Prognosis. Characteristics of the intervention: This retrospective analysis was conducted on the data of a prospective multicenter study launched by the International Brain Injury Association Disorders of Consciousness-Special Interest Group (IBIA DoC-SIG), aimed at examining the clinical evolution of a large sample of patients with pDoC and at identifying prognostic factors of outcome for the latter.	Clinical Diagnosis,l Consciousness Domain Index (CDI), Coma Recovery Scale (CRS-R)	Multivariate LR were used to examine the relationships between five independent variables (CDI, clinical diagnosis, CRS-R8, CRS-R10, and CRS-R total score) and patient outcomes at 6, 12, and 24 months post-injury, resulting in 15 models. Each regression included confounding variables such as age, gender, time since injury, and etiology. Model performance was evaluated using Nagelkerke's pseudo-R^2 to measure explained variance, and the area under the ROC curve (AuROC) to assess discriminatory ability.	The present validation study demonstrated that the CDI, an unsupervised machine-learning clinical index based on a combination of the different functions assessed by the CRS-R subscales, can improve prediction accuracy and sensitivity for consciousness recovery at 6, 12, and 24 months after brain injury.
(Zheng et al., 2022)	In this study, a DL model for neurological outcome prediction is proposed that leverages trend information in continuous EEG data to improve outcome prediction in comatose patients following cardiac arrest.	1038 patients with cardiac arrest and in a comatose state.	Intervention category: Prognosis. Characteristics of intervention: In this study, a DL model for neurological outcome prediction is developed that leverages trend information in continuous EEG data to improve outcome prediction in comatose patients following cardiac arrest. The performance of the proposed model is evaluated on a large international multicenter cardiac arrest EEG dataset developed to obtain a large and diverse cohort. The cardiac arrest EEG monitoring protocols were initiated during hypothermia of the patients and continued even after rewarming.	EEG	A time-dependent deep learning model using Bi-LSTM neural networks was developed to capture temporal patterns in EEG time series. Bi-LSTM processed the data in both forward and backward directions to learn dependencies over time. The final output of the top Bi-LSTM layer was passed to a fully connected layer, with dropout applied to prevent overfitting, and a softmax layer used to compute outcome probabilities. The model was trained using cross-entropy loss and the stochastic gradient descent with momentum (SGDM) optimizer.	Our results demonstrate that a deep learning model that leverages EEG dynamics can provide accurate neurologic outcome predictions post-cardiac arrest that become more accurate as time passes. Our time-sensitive models accuracy continued to increase as additional EEG data was included, reaching maximum predictive accuracy at 66 hours (AUC 0.88).
(Campagnini et al., 2024)	This study aimed to evaluate which information derived from the CRS-R provides the most reliable prediction of both the clinical	171 individuals with DoCs (82 UWS, 65 MCS+, 24 MCS-) were admitted to an inpatient	Intervention category: Prognosis. Characteristics of the intervention: The baseline neurobehavioral status of all participants was determined in the first two weeks of admission based on the results of five tests using the Spanish adaptation of the CRS-R.	Demographic and clinical data of individuals with DoC and CRS-R	ML classifiers were developed using seven datasets to predict clinical diagnosis and recovery of consciousness. For each outcome, four algorithms, LR, SVM, RF, and KNN, were implemented. Classifier hyperparameters were tuned using a 5×12-fold nested cross-validation. Models predicting clinical diagnosis at discharge	Recovery of full consciousness was predicted more accurately and similarly by all measures studied, except for initial clinical diagnosis. Both the total score in the CRS-R and the scores in the CRS-R subscales had the highest predictive value for predicting recovery of consciousness. In

	diagnosis and recovery of consciousness at discharge of a long-term neurorehabilitation program.	neurorehabilitation program.	Thereafter, participants' status was assessed weekly until MCS release, discharge, or death. In addition, during their participation in the neurorehabilitation program, participants were medically monitored to avoid clinical complications, reduce agitation, and relieve pain, and were provided with daily sessions of personalized physical therapy and multimodal sensory stimulation based on their individual needs.		were optimized based on accuracy, while models predicting recovery of consciousness used balanced accuracy due to class imbalance, as significantly fewer patients regained consciousness.	particular, the scores in the CRS-R subscales, together with the Consciousness Domain Index, obtained the best results in terms of accuracy and F1 score. The AIC and BIC values confirmed a higher model fit of the models with the emergency outcome compared to the clinical diagnosis at discharge. The similarity between the models with the different measures was also confirmed, presenting a reduced variability of the median values of AIC and BIC compared to those on the clinical diagnosis outcome.
(Riganello et al., 2010)	The aim of this study is to identify significant changes in heart rate variability (an emerging objective descriptor of autonomic correlates of brain activation) in response to complex emotionally charged auditory stimuli (music) through the application of data mining procedures.	Patients involved: 9 patients in vegetative state, 16 healthy volunteers.	Intervention category: Diagnosis. Characteristics of the intervention: VS subjects and controls were seated in a chair with constant room temperature and no transient noise. After resting baseline recording before each music sample, subjects were exposed binaurally to the four selected music samples, presented via earplugs, balanced for volume, and played in random sequence to minimize carryover effects. At the end of each music sample, healthy controls were asked to report and classify the emotions they had experienced. The distribution of emotions expressed for each music sample was determined and emotional responses were grouped as "positive" or "negative". Finally, heart rate was recorded via photoplethysmographic sensor.	Autonomic parameters: HRV	Data mining techniques were applied to the healthy controls dataset to extract meaningful patterns, such as general trends and associations between variables, that are novel, implicit in the data, and potentially useful for prediction and decision-making. The open-source software WEKA was used to train decision trees and identify association rules. Reported emotions for each musical sample served as labels, and the classification process aimed to balance recognition (accuracy on training data) with generalization (accuracy on test data). A 1-R rule-based system was used to classify emotional states, which were treated as target variables predicted solely from HRV parameters..	The results obtained from this study suggest that autonomic changes with possible emotional valence can be induced by complex stimuli even in a vegetative state, with implications on the residual reactivity of the subjects.
(Liuzzi et al., 2022)	The study aimed to investigate the impact of medical complications on clinical outcome prediction using machine learning models.	Multi-center, Longitudinal, Retrospective, Cohort, Observational.<br><br>Patients involved: 176 patients with pDoC (91 UWS, 85 MCS)	Intervention category: Prognosis. Characteristics of the intervention: Data from a large cohort of patients with pDoC enrolled in a multicenter, observational, longitudinal design were retrospectively analyzed. Furthermore, MCs that occurred in the first months of hospitalization in neurorehabilitation were assessed by direct clinical observation of hospital staff and grouped into 10 categories, with relative severity rating.	Demographics (age, sex), medical history (injury timing, etiology), the best total and sub-scores out of at least five. CRS-R evaluations, the level of functional disability indexed by the Disability Rating Scale total score (DRS), the level of clinical complexity as measured by the Early Rehabilitation Barthel Index (ERBI),medical comorbidities before the brain injury as assessed by the Cumulative Illness Rating Scale (CIRS),GOSE.	Admission data were analyzed using 5-fold cross-validation to compare the performance of four models: Elastic-Net (EN), Orthogonal-Matching Pursuit (OMP), KNN, and Support Vector Regression (SVR). Hyperparameters were tuned to minimize cross-validation error, with the 6-month GOS-E score as the target. To address class imbalance, training sets were resampled using the Synthetic Minority Oversampling Technique (SMOTE).	The proposed framework based on ML (with accessible features and without instrumental requirements) has demonstrated that the functional outcome of pDoC patients at 6 months from injury can be predicted at the time of admission with an accuracy of 88.6%. Furthermore, by performing an adjustment action of the model itself with information on MC it is possible to reach a prediction accuracy of 92.6%.
(Lee et al., 2022)	This study proposes an explainable	6 healthy patients in sleep state, 16	Intervention category: Prognosis.	Perturbational	A CNN model was trained to classify arousal and awareness levels as low or high. Domain	ECI effectively differentiates between low and high states of arousal and awareness in

	consciousness indicator (ECI) using deep learning to simultaneously distinguish and quantify the two components of consciousness, arousal and awareness, using a Convolutional Neural Network (CNN).	healthy patients under general anesthesia, 34 patients with severe brain injury (6 UWS, 10 MCS).	Characteristics of the intervention: For the sleep stage dataset, sleep stages were scored manually using the American Academy of Sleep Medicine Scoring Manual. When the participant entered a specific sleep stage, TMS was applied to the parietal cortex using a navigated brain stimulation system. Participants were subsequently awakened by an alarm sound after each session and were then asked if they had had a conscious experience.<br>For the anesthesia stage dataset, TMS was applied to the left parietal or motor regions after participants reached deep unresponsiveness. Additionally, upon awakening from anesthesia, reports of conscious experience during anesthesia were collected.<br>For the severe brain injury dataset, all patients in question had lapsed into a coma due to brain injury and had a prolonged state of altered consciousness. Relevant experts performed repeated CRS-R for each patient, including on the day of the TMS-EEG examination and before the fluorodeoxyglucose positron emission tomography (FDG-PET) scan.	complexity index (PCI), resting-state EEG.	similarity was first calculated to aid source domain selection for transfer learning. The CNN architecture included five convolutional layers with 2D filters, max-pooling, and a final softmax layer for classification. Parameters were learned via backpropagation, using ReLU as the activation function. For comparison, LDA and SVM with a polynomial kernel were also tested as baseline classifiers on the same data.	TMS-EEG results during sleep, anesthesia, and disorders of consciousness (DoC). The findings suggest that ECI could be applied to resting EEG data without TMS, maintaining accuracy. Its high correlation with PCI confirms its reliability using TMS-EEG. The consistency of independently derived measures supports deep learning as a valid approach. Additionally, CNN outperforms LDA and SVM in classifying arousal and awareness states, highlighting the framework's suitability for EEG data with nonlinear features.
(Wielek et al., 2018)	The aim of this study was to investigate the utility of a multivariate machine learning technique based on permutation entropy, a measure of complexity.	23 patients with DoC (12 UWS, 11 MCS) and 26 healthy participants	Intervention category: Diagnosis.<br>Characteristics of the intervention: The 24-hour longitudinal recording of each DoC patient was divided into light periods, which correspond to the circadian day and circadian night. Twenty-three video recordings were visually screened and scored into periods of "eyes closed" and "eyes open". Periods in which the state of the eyes repeatedly switched between open and closed were scored as open-closed.	Long term PSG	Unsupervised Model:<br>Hierarchical agglomerative clustering, a bottom-up approach, was applied to group similar n-dimensional feature vectors (epochs) into hierarchical clusters based on dissimilarity. The goal was to identify sleep-related patterns across healthy subjects, MCS patients, and UWS patients. Clustering was conducted separately for each group, using average epoch dissimilarities to form data-driven groupings without predefined labels.<br><br>Supervised Model:<br>Epoch-wise supervised classification was conducted at the individual level. A classifier was trained and validated using healthy subjects' data with standard sleep staging, applying a one-subject-excluded cross-validation approach. Two classifiers, Feedforward Neural Networks and RF, were compared.	The results of the study demonstrate that the classifier trained to identify 5 sleep stages on healthy data successfully generalized to a DoC dataset yielding a mean (i.e., across-subjects) accuracy of 0.63 (F-score), including 11 patients with high performance of 0.87 (F-score). Such cross-generalization, where the classifier was trained on data from one group and successfully applied to other data, indicates a correspondence between neural patterns in these groups, suggesting that the adopted highly data-driven approach to classify sleep/wake periods in DOC patients could represent an alternative to the current coarse and highly subjective estimation of sleep stages based on eye state or subjective criteria that are poorly consistent across publications.

					Hyperparameters were either fixed or optimized through grid search, using an additional 10-fold cross-validation on the training set.	
(Liu et al., 2023)	This study aimed to predict the correlation between consciousness and PBH by retrospectively collecting and analyzing computed tomography (CT) signs combined with artificial intelligence (AI) technique.	Retrospective.<br><br>Patients involved: 120 patients with brainstem hemorrhage (50 conscious, 26 MCS and 44 in coma).	Intervention category: Prognostic: Characteristics of the intervention: Transverse craniofacial CT scans were performed in all patients with orbitomeatal baselines as baselines and a scan range from skull apex to skull base. Consciousness was assessed using the Coma Recovery Scale-Revised (CRS-R) and based on literature. Hematomas were also analyzed using a categorical scale based on the classification of hematoma shape and hematoma density.	Gender, age, stages of intracerebral hemorrhage, CT signs with AI or radiology physicians, hemorrhage involving the midbrain or ventricular system.	The raw CT images were analyzed in DICOM (digital imaging and communications in medicine) format for all cases of cerebral hemorrhage using the auxiliary diagnostic software DEEPWISE Medical Artificial intelligence, thus collecting the hemorrhage volumes and CT values. Specifically, the AI system calculated the hemorrhage volumes and then directly extracted the mean CT values by CT tool. Then, to find factors that could indicate the state of consciousness, an ordered multivariate logistic analysis was performed on the statistically significant variables. To meet the conditions of the ordered multivariate logistic analysis, the continuous variables were grouped in order. Finally, to find out the relationships between hemorrhage volumes and the state of consciousness in more detail, the ROC curve was plotted.	The study demonstrated that CT signs combined with AI can predict the correlation between consciousness and PBH, and that hemorrhage volume and hemorrhage involving the ventricular system are two independent factors, with hemorrhage volume in particular achieving quantitative predictions.
(Di Gregorio et al., 2022)	The aim of the study was to examine the accuracy of clinical EEG-based psychophysiological biomarkers in predicting clinical outcomes in DoC patients.	Retrospective.<br><br>Patients involved: 33 DoC patients with traumatic and non-traumatic etiologies.	Intervention category: Diagnosis. Characteristics of the intervention: Electrophysiological measures were extracted from a standard clinical EEG recorded 1 month after acute brain injury and then used to discriminate the etiology of brain injury and predict clinical outcome 6 months after injury in DoC patients. A total of four quantitative EEG (qEEG) measures were extracted: z-score power spectral density, dominant frequency peak, permutation entropy, and mean amplitude.	Glasgow Outcome Scale (GOS), quantitative EEG (qEEG) [z-scored power spectral density, dominant frequency peak, permutation entropy, and mean amplitude], EEG-based functional connectivity	A stepwise linear discriminant analysis (LDA) with leave-one-subject-out cross-validation was applied. The goal of LDA was to discriminate two classes of data in a low-dimensional space while retaining the features with the highest discriminating power. Within leave-one-subject-out cross-validation, each feature matrix was used once as validation data, with the remaining data as training data, and then the percentage of correctly classified instances was calculated. The latter represents the classification accuracy. For classification, clinical outcome (i.e., improved vs. non-improved patients) was used as a categorical grouping variable and etiology (i.e., TBI vs. non-TBI) was used as a factor. In this way, the accuracy of EEG biomarkers of clinical outcome was estimated separately for TBI and non-TBI patients. Given the retrospective nature of the study, it was possible to handle unbalanced classifiers, thus having access to the calculation of additional metrics, such as balanced accuracy (the average of sensitivity and specificity) and precision (the number of positive class predictions divided by the sum of true-positive and false-positive instances). Only EEG variables that showed sensitivity to distinguish between groups in the analyses performed to address the first step entered the LDA.	This study presented three main findings. The most notable finding allowed to describe the advantages and limitations of standard EEG as a tool for clinical assessment and classification of DoC patients after severe acquired brain injury. Second, standard EEG-based measures (such as qEEG and functional connectivity) are promising tools to predict and classify DoC patients in terms of rehabilitation potential and functional recovery. Finally, the results demonstrated that different measures extracted from standard EEG could be fruitfully combined to discriminate the potential clinical outcome of patients using a machine learning approach. Thus, EEG biomarkers could prove to be highly relevant in supporting clinical and rehabilitation decision-making.

(El-Rashidy et al., 2023)	The primary objective of this study is to introduce the edge/cloud system to improve the efficiency of consciousness measurement through efficient local data processing and also propose an efficient machine learning (ML) model to predict the consciousness level of a given patient based on the patient's demographics, vital signs, and laboratory tests.	Patients involved: 10349 patient records were extracted from the entire Medical Information Mart for Intensive Care III (MIMIC-III) dataset, and used to train/test the model to predict the level of consciousness.	Intervention category: Diagnosis.<br><br>Characteristics of the intervention: The proposed edge/cloud system framework comprises three phases: vital sign acquisition, Fog-Assisted Consciousness Management (FACM), and cloud-based clinical service delivery. Vital sign acquisition gathers data from IoT sensors, wearable devices, and medical reports. FACM manages data transmission between the cloud and end users during treatment and prevention. Cloud computing enables secure storage, processing, and analysis of revised vital data, facilitating healthcare digitalization.	Glasgow coma scale (GCS), vital signs information and medical report informations.	The machine learning models used in this study are Linear Regression (LR), Support Vector Regressor (SVR), Decision Tree (DT), K-Nearest Neighbors (KNN), Ridge, Gradient Boosting Regressor (GBR) and Random Forest (RM). Feature selection is performed based on the importance of the features and their impact on the output based on their correlation with the output. Simulation results showed that the proposed SVM, KNN and RF models achieved the best performance before GCS value prediction. Those models without feature selection achieved a performance score of 0.835, 0.849 and 0.923 for KNN, SVM and RF respectively. However, with feature selection they managed to achieve a performance score of 0.934, 0.929 and 0.946 for KNN, SVM and RF respectively.	Simulation results reveal that the proposed ensemble models exhibit superior performance before consciousness level prediction. Therefore, it can be considered as an efficient solution for consciousness level prediction in IoT and cloud/edge environments.
(Molteni et al., 2019)	The aim of this study was to apply ML techniques to neurobehavioral data obtained from multiple subjects to derive a prediction model that can be applied to formulate a prognosis for individual new pediatric patients admitted with DoC, perform a multi-class prediction on four labels, and understand which domains of the neurobehavioral assessment contribute most to the prognostic decision.	Patients involved: The sample consisted of 124 patients diagnosed with UWS or MCS (and who presented with severe acquired brain injury of traumatic, anoxic, vascular or infectious etiology).	Intervention category: Prognosis.<br>Characteristics of the intervention: The LOCFAS assessments included in this study were<br>performed at 3 months (T0) and 6 months (T1) after admission.<br>Neurobehavioral characteristics were extracted for both assessments. Prognostic information at 4 levels was recorded 5 years after admission (death=0, UWS=1, MCS=2, exitMCS=3). Experiments were performed for each classifier in two modes: for prognostic prediction at 3 (T0) and 6 months (T1) after admission. For prediction at T0, only clinical and neurobehavioral characteristics at 3 months were provided as input for each classifier. For predictions at T1, all characteristics (i.e. clinical and neurobehavioral at T0 and T1) were included.	Neurobehavioural features of Level of Cognitive Functioning (LCF)	Four models (Random Forest, Logistic Regression, Support Vector, Gaussian Processes-based) were implemented to compare for classification. For each model, the dataset was split into three parts: 40% training, 40% testing and 20% hold-out set. The Random Forest (RF) classifier, based on an ensemble of decision trees, was run for CV with 1000 estimators, after checking the stability of the out-of-bag error rate and results. The Logistic Regression (LR) classifier was run on the data with intercept_scaling=10 and all other parameters set to default, including penalty=l2 and one-versus-rest decision strategy. The Linear Support Vector (SV) classifier was optimized<br>and run with error penalty C=1, tolerance threshold 0.0001 and<br>one-versus-rest decision strategy. The Gaussian process (GP) based classifier with radial basis function as kernel and multi-class one-on-one classification was optimized and performed.	This study demonstrated that multi-class prognostic prediction of pediatric DoC using ML could be feasible after 6 months from the event, if a large database (>100) of known pediatric cases, including neurobehavioral data, is available.
(Wang et al., 2022)	This study proposes a computer-aided approach for automatic detection of DoC by extracting information from electroencephalogram (EEG) signals, introducing a novel connectivity measure (the power spectral	Patients involved: 607 subjects suffering from brain injuries (including cerebral infarction, cerebral hemorrhage, intracranial infection and epilepsy)	Intervention category: Diagnosis.<br>Characteristics of the intervention: Clinical examinations with assessments of consciousness including pain response, arousal reaction, spontaneous acts, tendon reflex, light reflex and vital signs were performed immediately before recording the EEG signals. Furthermore, all clinical assessments were performed by physicians, who	Power Spectral Density Difference (PSDD) incorporating with a recursive Cosine function (CPSDD)	In this study, an EOSVM consisting of multiple support vector machines (SVM) classifiers is designed. Each of the SVMs is a binary classifier for classifying a subject to the DoC (+) class or the awake (−) class. The number of SVMs in the EOSVM classifier,N, should be adjusted based on the distribution of the original dataset. Typically, the more heavily unbalanced the dataset, the more SVMs should be used in the EOSVM classifier. In the experiments of this study, 100 SVMs are incorporated into the	The classification results of the study show that the EOSVM classifier with the new CPSDD connectivity measure achieved the best classification performance among 12 connectivity measures. For a setting of 97% majority vote from all SVMs, the EOSVM diagnosed, with high confidence, 35% of the patients with accuracy, sensitivity and specificity of 98.21%, 100% and 95.79%, respectively. Therefore, the EOSVM classifier incorporating the new CPSDD

	density difference [PSDD] incorporating a recursive cosine function [CPSDD]), and classifying brain-lesioned patients into DoC (i.e., positive) and awake (i.e., negative) classes via an ensemble of multivariate support vector machines (EOSVMs).		were blinded to the subjects' EEG connectivity measures.		EOSVM classifier. The EOSVM framework is characterized with a training phase and a testing phase. In the training phase, the EOSVM is first trained by the training data. Then, it is tested using the testing dataset in the testing phase. The whole training and testing process is performed in three phases: splitting the data into training and testing, classification in training and testing, and voting for the final results in testing.	connectivity measure is a promising tool for automatic detection of DoC in brain lesions.
(Zheng et al., 2017)	In the present study, probabilistic diffusion tractography is used to assess the structural connectivity between the thalamus and the cortex in VS, MCS− and MCS+ patients and to identify connectivity differences that could be used as reliable biomarkers for stratifying DOC patients.	Sample size of 25 patients with 10 VS (4T/6NT), 7 MCS− (6T/1NT) and 8 MCS+ (7T/1NT).	Intervention category: Diagnosis. Characteristics of the intervention: Using the tools of the FSL diffusion toolbox (FDT), a probability distribution function was estimated on the main direction of the fibers in each voxel. The next two approaches applied were: calculating the connection probability from the thalamus to each cortical target (where each voxel within the thalamus was quantified by the total number of samples reaching a target) and creating a path distribution map from the left or right thalamus (i.e., reconstructed thalamic traces) to identify all paths originating from the thalamus and projecting throughout the entire brain, including cortical targets. Both outputs from the two approaches were then used in the multiturn analysis, where each of the seven thalamo-cortical connectivity clusters was compared between VS and MCS+, VS and MCS−, and MCS− and MCS+.	Structural connectivity in various thalamo-cortical circuits	Multivariate classification techniques were used to test the reliability of neural markers. A reflector mapping method helped identify which areas along thalamic pathways showed the most consistent differences between patient groups. For each voxel in the brain, a 5-voxel sphere was created, and the thalamic connectivity values within each sphere were used as features in three binary SVM classifications: MCS+ vs. UWS, MCS− vs. UWS, and MCS+ vs. MCS−. Classification accuracy was calculated using a leave-2-subjects-out cross-validation to avoid bias. Each voxel was assigned the accuracy score of its surrounding sphere, producing whole-brain accuracy maps. Statistical significance was determined by comparing results to a random distribution generated through label shuffling.	It has been shown that the structural connectivity of individual thalamo-cortical networks and the preservation of this system in general can explain the gradations of consciousness observed in these severely brain-injured patients and that the combination of probabilistic tractography with searchlight classification presents a novel approach to the identification of biomarkers that could complement existing behavioral assessments and aid in differential diagnoses.
(Narayanan et al., 2023)	The aim of this study is to apply ML techniques to predict PDOC diagnostic states from variables obtained from two noninvasive neurobehavioral assessment tools and to apply network analysis to guide potential intervention strategies.	Sample of 74 patients with PDOC persisting for more than 4 weeks.	Intervention category: Diagnosis. Characteristics of the intervention: MATADOC and CRS-R data were collected simultaneously for each patient on four occasions over a 2-week period by trained clinical raters (blinded to the results of the other measure on each occasion). The MATADOC data for this study consisted, for each occasion, of the five main subscale items (responses to visual stimuli, responses to auditory stimuli, awareness of musical stimuli, responses to verbal	Coma Recovery Scale-Revised (CRS-R), Music Therapy Assessment Tool for Awareness in Disorders of Consciousness (MATADOC)	In this study, artificial neural networks (feedforward with weight adjustments) and decision trees are used for supervised learning, while cluster analysis is used for unsupervised learning. Artificial neural networks in the form of perceptrons (models with weight refinement by repeated presentation of the data, along with additional levels of weights by "hidden" levels of nodes) and decision trees in the form of J48 (a tree-generating algorithm based on a top-down strategy to find, at each level of the tree, the attribute or attributes with the most information to split the samples into the specified class values) are used for supervised learning using	This study demonstrated that ML can derive rules for diagnosing PDOC with data from two neurobehavioral tools without the need to collect large clinical and imaging datasets. Indeed, using only 13 variables along with computed and induced diagnostic states resulted in models that, with cross-validation, were shown to be accurate enough to warrant further investigation in clinical settings.<br>In summary, network analysis using measures obtained from these two noninvasive tools provides new systems-level ways to interpret possible transitions

			commands, and arousal), the two secondary scale items (behavioral response to music and musical response), plus a diagnostic coma status for that occasion calculated (in accordance with the measurement protocol) by summing the five main items. Additionally, each patient was assigned four sets of CRS-R data (the latter consisting of 6 functional subscales [FS] in the auditory, visual, motor, oromotor [verbal], communicative, and arousal domains) obtained through sessions lasting 15–30 minutes.		10-fold cross-validation. Unsupervised learning, on the other hand, occurs via k-means clustering, where samples in one cluster should have more in common with each other than they do with samples in another cluster.	between PDOC states, leading to potential use in novel next-generation decision support systems for PDOC.
(Muller et al., 2019)	The aim of the study was to apply predictive models using machine learning methods to examine, one year after onset, the prognostic power of serial NSE measurements in patients with AI-DOC and to compare the discriminative accuracy of this method with standard absolute single-day and relative day-to-day difference NSE levels.	Data from a total of 143 patients analyzed	Intervention category: Outcome prediction, Predictive.<br>Characteristics of the intervention: Blood sampling was performed for NSE measurement. Samples were collected after admission (day 0) and on subsequent mornings at approximately 6:00 AM as part of the daily routine. NSE measurements were performed at irregular intervals for each patient, with a median of 3 measurements within the first 18 days. In addition, neurological outcome was assessed using the Clinical Performance Category (CPC). The scale classified patients into the following categories: dead, vegetative state, severe disability, moderate disability, and good recovery.	Serum neuron-specific enolase (NSE)	In the present study, the classification algorithms employed were: logistic regression (LR), that allow a binary classification of the data using a logistic function, support vector machines (SVM), that define a straight line decision boundary in feature space, nearest neighbor clustering (KNN), that classify each patient based on the class belonging to the majority of their nearest neighbors within a feature space, and naive Bayesian classification, that aims to maximise the likelihood of the posterior distribution, assumed to be independent for each NSE variable.	Through the obtained results, this study suggests that predictive models for NSE prognosis could provide an additional tool for accurate outcome prediction for a challenging clinical population, since the models applied to serial measurements provided risk score estimates, unlike the single and relative days cut-off approach.
(Campbell et al., 2020)	The aim of the study was to further improve the understanding and diagnosis of DoC by systematically comparing popular machine learning approaches to classification and to evaluate a novel source of model training data, namely participants in anesthesia-induced unconsciousness.	83 subjects were scanned and divided into 3 data sets: propofol and sevoflurane anesthesia, propofol anesthesia, and DoC patients (13 UWS, 8 MCS).	Intervention category: Diagnosis.<br><br>Characteristics of the intervention:<br>Data Set 1: Subjects in this data set received propofol anesthetics with light sedation and general anesthesia, during which intravenous propofol anesthetic was infused through an intravenous catheter inserted into a vein in the right hand or forearm. Behavioral responsiveness was assessed using the Ramsay scale. Rs-fMRI data acquisition consisted of three 8-minute scans in baseline wakefulness, light sedation, and general anesthesia.	Resting-state fMRI,	This study evaluated three types of learning models:<br><br>SVM: A model that separates two classes by creating a boundary (hyperplane) that maximizes the distance between them in multi-dimensional space.<br>Extra Trees (ET): A type of decision tree model that adds randomness when choosing where to split the data. It builds many trees and combines their results, making it more robust.<br>Artificial Neural Network (ANN): A model made up of layers of connected "neurons" that adjust their connections based on errors. In this study, a simple feedforward network (multilayer perceptron) was used, with an input layer, two hidden layers, and one output node.	The findings from this study demonstrate that machine learning classifiers trained on rs-fMRI features derived from participants under anesthesia have the potential to aid discrimination between degrees of pathological unconsciousness in clinical patients.

			Data Set 2: Subjects in this data set received propofol sedation. Four behavioral response conditions were determined by the OAAS score, namely wakefulness baseline, light propofol sedation, deep propofol sedation, and recovery. Rs-fMRI data acquisition consisted of four 15-minute scans in baseline wakefulness, light and deep sedation, and recovery.<br>Data Set 3: Subjects in this data set were assessed using a standardized behavioral assessment, the CRS-R, on the day of the fMRI scan, both before and after the scan. For Rs-fMRI data, two hundred EPI volumes (6 minutes and 40 seconds) were acquired, as well as high-resolution anatomical images.			
(Yang et al., 2024)	This study aimed to develop a cascade 3D EfficientNet-B3-based deep learning framework (named DeepDOC) that uses preprocessed rsfMRI images to distinguish MCS patients from UWS patients.	Cohort, Retrospective.<br><br>Patients involved: Initial Data Set; 140 patients, including 76 UWS, 25 MCS, and 39 controls. Independent Data Set: 11 patients (4 with cognitive-motor dissociation and 7 without it).	Intervention category: Diagnosis.<br>Characteristics of the intervention: During rs-fMRI scans, healthy subjects were asked to close their eyes, relax while remaining alert, and refrain from any structured thinking. Depending on the recruitment site (hospital), MR images were acquired using a Philips Ingenia 3 Tesla, GE Signa 3 Tesla, or Siemens 3 Tesla scanner. Functional images were acquired using a T2*-weighted EPI sequence, each with its own specifications based on the scanner used. High-resolution, three-dimensional T1-weighted gradient ultrasound images were also obtained for each participant to facilitate registration and localization of functional images.	Resting-state functional MRI (rs-fMRI)	Because 3D-CNNs are very good at handling both spatial and temporal information, a new model called DeepDOC was created using two connected 3D EfficientNet-B3 networks. The first network separated DoC patients from healthy controls. The second network then classified DoC patients into subgroups like MCS vs. UWS or CMD vs. non-CMD. This system also produced neuroimaging markers linked to conscious states.<br>DeepDOC was compared to five standard machine learning models:<br>SVM: Finds the best line (or surface) to separate groups.<br>Logistic Regression: Estimates the chance of a certain outcome.<br>Random Forest: Uses many decision trees to make predictions.<br>XGBoost: Builds a series of models that fix previous errors.<br>AdaBoost: Combines simple models into a stronger one by adjusting their importance.	The study results demonstrate that DeepDOC outperforms the five machine learning models, achieving an area under the curve (AUC) of 0.927 and an accuracy of 0.861 to distinguish MCS and UWS patients. More importantly, DeepDOC excels in identifying CMD, achieving an AUC of 1 and an accuracy of 0.909. Using the gradient-weighted class activation mapping algorithm, we found that the posterior cortex, which includes the visual cortex, posterior. In conclusion, showing high accuracy, interpretability, and generalization ability, DeepDOC provides a valuable end-to-end solution for the differential diagnosis of these OCD subgroups in the clinical setting.

Legend: AI – Artificial Intelligence; ML – Machine Learning; DL – Deep Learning; LMM – Linear Mixed Models; EEG – Electroencephalogram; ECG – Electrocardiogram; SVM – Support Vector Machine; LR – Logistic Regression; K-NN – K-Nearest Neighbors; DT – Decision Tree; RF – Random Forest; GBT – Gradient Boosting Trees; XGBoost – eXtreme Gradient Boosting; MMN – Mismatch Negativity; ERP – Event-Related Potential; LFS – Localized Feature Selection; LOSO – Leave-One-Subject-Out; CNN – Convolutional Neural Network; GCS – Glasgow Coma Scale; CPC – Cerebral Performance Category; CDI – Consciousness Domain Index; CRS-R – Coma Recovery Scale-Revised; Bi-LSTM – Bidirectional Long Short-Term Memory; SGDM – Stochastic Gradient Descent with Momentum; DoCs – Disorders of Consciousness; AIC – Akaike Information Criterion; BIC – Bayesian Information Criterion; HRV – Heart Rate Variability;WEKA – Waikato Environment for Knowledge Analysis; pDoC – Prolonged Disorders of Consciousness; DRS – Disability Rating Scale; ERBI – Early Rehabilitation Barthel Index; CIRS – Cumulative Illness Rating Scale; GOSE – Glasgow Outcome Scale – Extended; EN – Elastic-Net; OMP – Orthogonal Matching Pursuit; SVR – Support Vector Regressor; SMOTE – Synthetic Minority Oversampling Technique; ECI – Explainable

Consciousness Indicator; TMS – Transcranial Magnetic Stimulation; FDG-PET – Fluorodeoxyglucose Positron Emission Tomography; PCI – Perturbational Complexity Index ; LDA – Linear Discriminant Analysis; MCS – Minimally Conscious State; UWS – Unresponsive Wakefulness Syndrome; PSG – Polysomnography; CT – Computed Tomography; DICOM – Digital Imaging and Communications in Medicine; qEEG – quantitative Electroencephalogram; TBI – Traumatic Brain Injury; MIMIC-III – Medical Information Mart for Intensive Care II; IoT – Internet of Things; LOCFAS – Levels of Cognitive Functioning Assessment Scale ; LCF – Level of Cognitive Functioning; GP – Gaussian Process; PSDD – Power Spectral Density Difference; CPSDD – recursive cosine function; EOSVM – Ensemble of Support Vector Machines; VS – Vegetative State; MATADOC – Music Therapy Assessment Tool for Awareness in Disorders of Consciousness; NSE – Neuron-Specific Enolase; rs-fMRI – resting-state functional Magnetic Resonance Imaging; OAAS – Observer's Assessment of Alertness/Sedation; EPI – Echo-Planar Imaging; ET – Extra Trees; ANN – Artificial Neural Network; 3D-CNN – 3-Dimensional Convolutional Neural Network; SOTA – State Of The Art; AdaBoost – Adaptive Boosting.
